# Successful Percutaneous Coronary Intervention for Atherosclerotic Coronary Lesion with Anomalous Origin of the Right Coronary Artery

**DOI:** 10.1155/2018/4232941

**Published:** 2018-07-19

**Authors:** Junji Matsuda, Takashi Ikenouchi, Giichi Nitta, Shunichi Kato, Kazuya Murata, Miki Kanoh, Yukihiro Inamura, Nobutaka Kato, Tomomasa Takamiya, Ken Negi, Akira Sato, Tsunehiro Yamato, Yutaka Matsumura, Junichi Nitta

**Affiliations:** ^1^Department of Cardiovascular Medicine, Japanese Red Cross Saitama Hospital, Saitama, Japan; ^2^Department of Cardiovascular Medicine, Tokyo Medical and Dental University, Tokyo, Japan

## Abstract

Congenital coronary artery anomalies, including anomalous origin of a coronary artery, can manifest as life-threatening conditions, such as myocardial infarction or arrhythmia, and may even lead to sudden death associated with specific congenital anatomical features. Such arteries can also develop atherosclerotic lesions. This report describes the case of a 75-year-old man who was admitted to our hospital due to exertional dyspnea. The right coronary artery was found to originate from the left coronary sinus and exhibit tight stenosis due to atherosclerosis, causing effort angina pectoris. This case highlights the fact that coronary artery anomalies can cause angina pectoris via both atherosclerotic and nonatherosclerotic effects, and successful revascularization was achieved noninvasively via percutaneous coronary angioplasty.

## 1. Introduction

The incidence of congenital coronary artery anomalies has been reported at less than 1%, indicating that such anatomical abnormalities are relatively rare [1–3]. Such conditions are characterized by abnormal location of the orifice of a coronary artery, including anomalous aortic origin of a coronary artery (AAOCA). Sometimes, these abnormal anatomical features can lead to life-threatening presentations such as myocardial infarction or arrhythmia and even sudden death [4, 5]. Anomalous aortic origin of the right coronary artery (AAORCA) describes the abnormal location of the orifice of the right coronary artery (RCA) in the left coronary sinus, an abnormality shown to carry a risk of myocardial infarction and sudden death [6]. Such arteries can also develop atherosclerotic lesions. Although some studies have reported successful revascularization of these coronary abnormalities using surgical coronary bypass or percutaneous coronary angioplasty, the optimal revascularization strategy remains unclear. This report describes the case of a 75-year-old man with AAORCA and effort angina pectoris related to an atherosclerotic lesion in the RCA, which was successfully treated noninvasively using percutaneous coronary angioplasty.

## 2. Case

**Figure 1:**
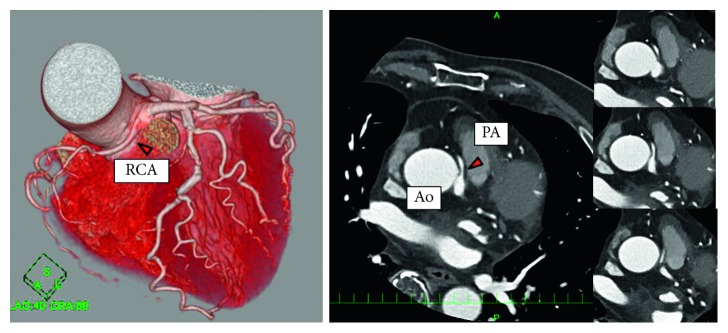
Coronary computed tomography revealing that the RCA originates from the left coronary sinus and passes between two great vessels. Ao = aorta, PA = pulmonary artery, and RCA = right coronary artery.

**Figure 2:**
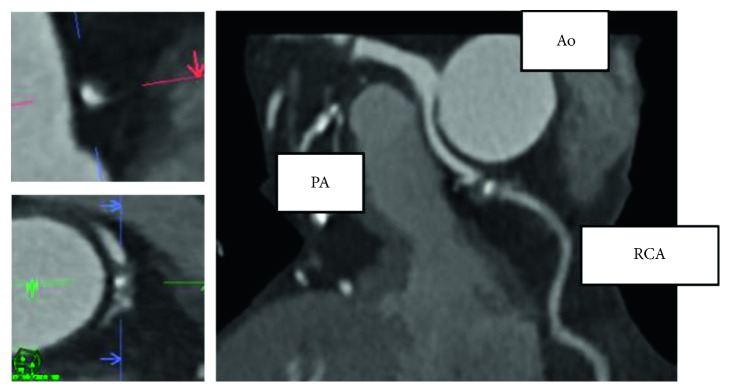
Atherosclerotic findings of the RCA lesion. Spotty calcification and mild positive remodeling were noted. Ao = aorta, PA = pulmonary artery, and RCA = right coronary artery.

**Figure 3:**
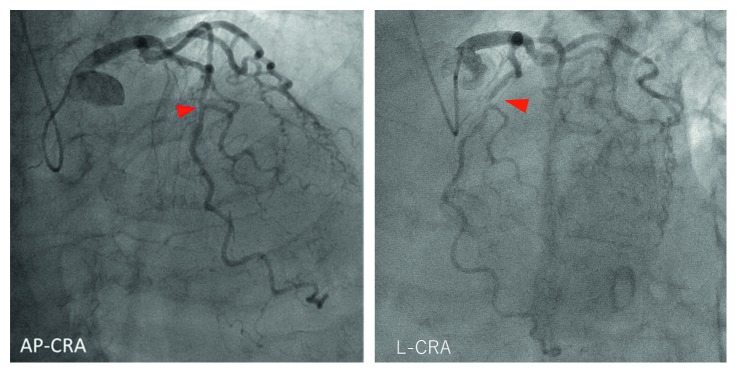
Coronary angiography revealing tight stenosis at the LAD-D1 bifurcation. AP = anterior-posterior projection, CRA = cranial angulation, D1 = first diagonal branch, L = lateral projection, and LAD = left anterior descending artery.

**Figure 4:**
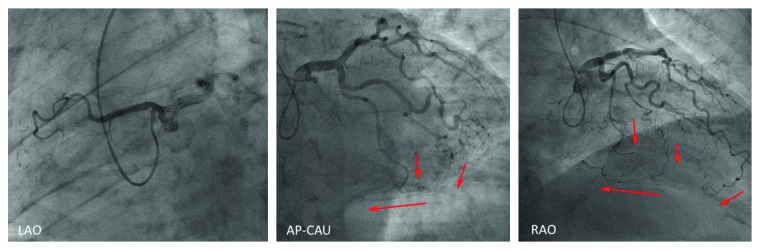
Coronary angiography revealing a completely occluded right coronary artery originating from the left coronary sinus. The abnormal right coronary artery had multiple collaterals from the left anterior descending artery and left circumflex branch. AP = anterior-posterior projection, CAU = caudal angulation, LAO = left anterior oblique view, and RAO = right anterior oblique view.

**Figure 5:**
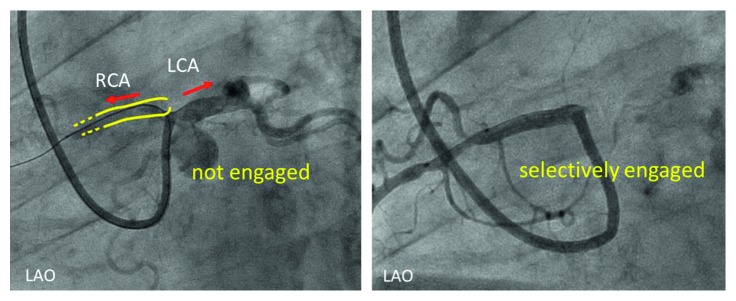
Cannulation of the guiding catheter to the anomalous orifice of the RCA. Cannulation was difficult, and it was not possible to achieve adequate backup support. A 7-Fr Amplatz Left II Mach 1 coronary catheter was used. LAO = left anterior oblique view, LCA = left coronary artery, and RCA = right coronary artery.

**Figure 6:**
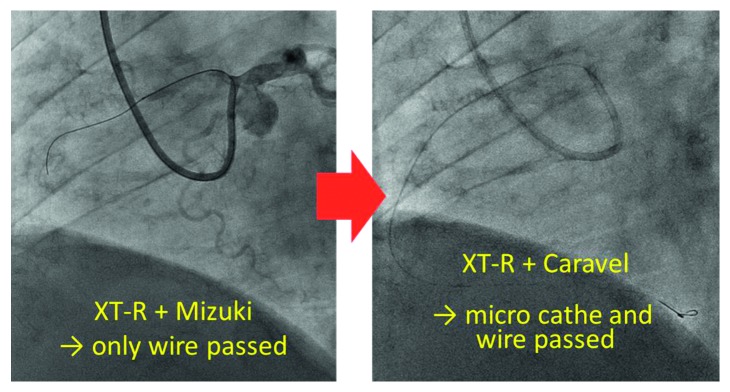
Passing the guide wire and microcatheter through the lesion. When using the XT-R guide wire, only the Caravel and not the Mizuki microcatheter could be passed though the lesion. The anchor balloon technique was used to create extra support for the guiding catheter. The wire was then exchanged to Sion blue.

**Figure 7:**
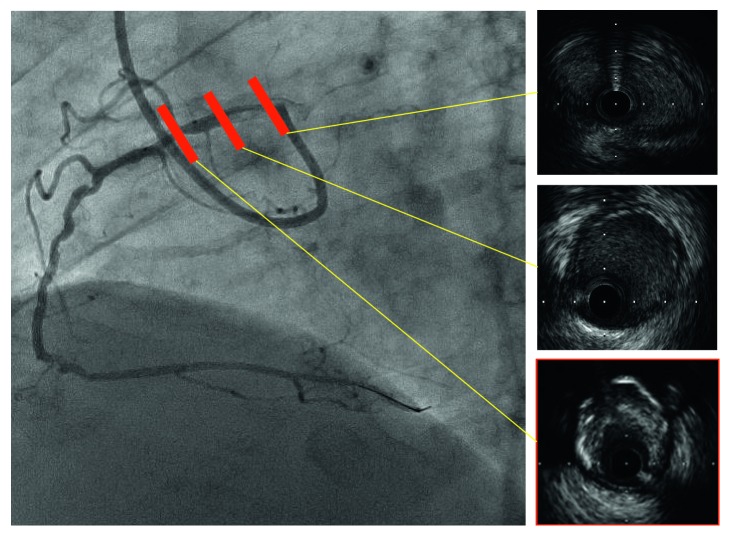
Intravascular ultrasound revealing diffuse eccentric calcified plaque, and the intramural course of the proximal ectopic artery was elliptical with some lateral compression.

**Figure 8:**
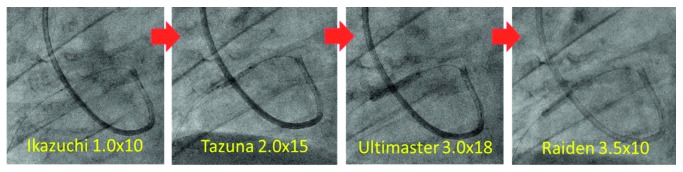
Stenting strategy. After preballooning with Ikazuchi 1.0 × 10 mm and Tazuna 2.0 × 15 mm, angioplasty with a drug-eluting stent (Ultimaster 3.0 × 18 mm) was performed, followed ballooning with Raiden 3.5 × 10 mm.

**Figure 9:**
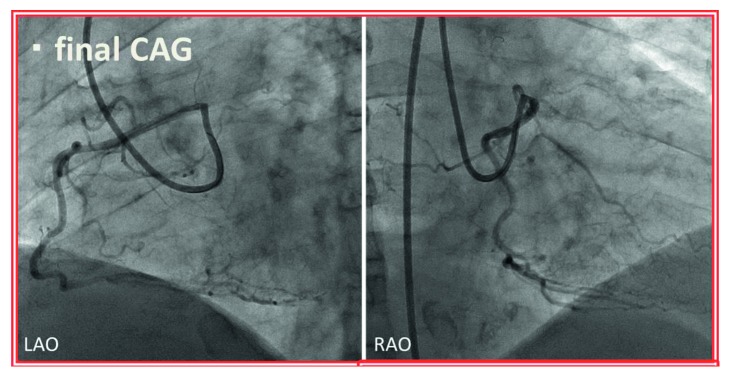
Final coronary angiography (CAG). Optimal results were noted. LAO = left anterior oblique view and RAO = right anterior oblique view.

**Figure 10:**
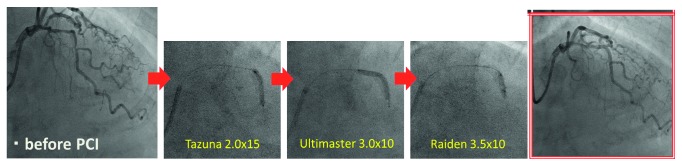
Percutaneous coronary intervention (PCI) for revascularization of the left anterior descending artery. After preballooning with Tazuna 2.0 × 15 mm, angioplasty with a drug-eluting stent was performed (Ultimaster 3.0 × 10 mm), followed by ballooning with Raiden 3.5 × 10 mm.

A 75-year-old man was admitted to our hospital due to exertional dyspnea that had been manifesting for several months. The patient had coronary risk factors, including hypertension, dyslipidemia, family history of coronary disease, and past history of smoking, and was taking medication prescribed by his regular physician (nifedipine 20 mg/day for hypertension; bezafibrate 400 mg/day for hyperlipidemia). On admission, physical examination and laboratory data revealed no specific findings: white blood cell count, 3990 cells/*μ*L; hemoglobin, 14.8 g/dL; platelets, 26.7 × 104 cells/*μ*L; glucose, 95 mg/dL; blood urea nitrogen, 14 mg/dL; creatinine, 0.71 mg/dL; estimated glomerular filtration rate, 81.7 mL/min; uric acid, 6.0 mg/dL; aspartate transaminase, 21 U/L; alanine aminotransferase, 15 U/L; total bilirubin, 0.6 mg/dL; creatine kinase, 85 IU/L; creatine kinase-MB, 8 IU/L; C-reactive protein, 0.1 mg/dL; low-density lipoprotein cholesterol, 150 mg/dL; high-density lipoprotein cholesterol, 63 mg/dL; triglycerides, 113 mg/dL; glycated hemoglobin, 5.4%; brain-type natriuretic peptide, 24 pg/mL. However, the exercise stress test revealed slight ST depression in leads V4-6 on electrocardiography. Based on the clinical symptoms, the patient was suspected of coronary artery disease. Adenosine triphosphate-stress radionuclide myocardial perfusion imaging revealed inferolateral wall ischemia. Coronary computed tomography indicated that the RCA originated from the left coronary sinus and passed between the aorta and the pulmonary artery (Figure 1). Total occlusion in the midportion of the abnormal RCA and 90% stenosis of the left anterior descending coronary artery (LAD) were suspected. The RCA lesion had atherosclerotic findings such as spotty calcification and mild positive remodeling (Figure 2). The patient was diagnosed with effort angina pectoris and underwent coronary angiography, which revealed tight stenosis at the LAD-D1 bifurcation and a completely occluded RCA originating from the left coronary sinus. The abnormal RCA had multiple collaterals from the LAD and left circumflex branch (Figures 3 and 4). Because the patient had a coronary artery anomaly and multivessel stenosis, coronary artery bypass grafting was proposed for revascularization, but the patient refused any surgically invasive treatment. Therefore, percutaneous coronary intervention (PCI) was performed for revascularization. The following revascularization systems were used: right femoral artery approach; guiding catheter, 7-Fr Amplatz Left 2 Mach 1 (Boston Scientific, Natick, MA, USA); guide wire, Grand Slam, XT-R, and Sion blue (both from Asahi Intecc, Aichi, Japan); microcatheter, Mizuki (Kaneka Medical, Osaka, Japan) and Caravel (Asahi Intecc); balloon, Ikazuchi 1.0 × 10 mm (Kaneka Medical), Tazuna 2.0 × 15 mm (Terumo Corporation, Tokyo, Japan), and Raiden 3.5 × 10 mm (Kaneka Medical); stent, Ultimaster 3.0 × 18 mm (Terumo Corporation); and intravascular ultrasound (IVUS) catheter, 40 MHz rotational OptiCross (Boston Scientific). Cannulating the guiding catheter to the anomalous orifice of the RCA was difficult, and it was not possible to achieve adequate backup support (Figure 5). Using the XT-R guide wire, the Mizuki microcatheter could not be passed though the chronic total occlusion lesion until it was replaced with a Caravel microcatheter. The wire was then changed to Sion blue (Figure 6). IVUS revealed diffuse eccentric calcified plaque. The intramural course of the proximal ectopic artery was elliptical with some lateral compression. However, the stenosis of the proximal intramural course was not so severe that we did not deploy the stent in the proximal portion (Figure 7). Multiple ballooning and angioplasty with a drug-eluting stent were performed (Figure 8), and an optimal result was obtained (Figure 9). At the same time, PCI was performed for LAD revascularization, and an optimal result was obtained (Figure 10). No exertional dyspnea was noted following PCI. At approximately one year after intervention, exercise stress radionuclide myocardial perfusion imaging and coronary angiography revealed no in-stent restenosis or ischemia. The patient expressed satisfaction with the outcome of the intervention.

## 3. Discussion

This case illustrates the possibility that coronary artery anomalies can be complicated with atherosclerotic lesions and can cause angina pectoris. PCI may represent a suitable option for the noninvasive treatment of such abnormal arteries.

AAOCA represents a congenital coronary artery anomaly in which the artery originates from the contralateral aortic sinus. Thus, AAORCA describes the abnormal location of the RCA orifice in the left coronary sinus. The mortality rates associated with AAOCA have been reported at 0–50% for RCA abnormalities and 30–100% for left coronary artery abnormalities [7]. The interarterial course of the RCA, which runs between the aorta and pulmonary artery (as in the present case), is considered malignant because it carries a risk of myocardial ischemia and sudden death even in the absence of atherosclerosis. Several mechanisms have been proposed to underlie the mechanism by which such abnormalities cause ischemia in the absence of atherosclerosis: compression of the anomalous artery between the aorta and pulmonary artery (especially during exercise), compression of the anomalous artery by the aortic commissure, and angulation of the coronary artery origin with increased aortic wall tension or intramural course [8, 9]. In contrast, some reports highlight the possibility that the proximal portion of the anomalous RCA could be more susceptible to atherosclerosis due to shear stress and vascular endothelium dysfunction caused by compression or by the anatomical feature itself [10]. Some reports of acute myocardial infarction with AAORCA indicated that the atherosclerotic culprit lesion was mostly seen at the proximal portion of the anomalous artery (75%). It is suspected that such anatomical abnormalities increase the risk of plaque formation and progression of atherosclerosis [11, 12]. In patients with coronary anomaly, both atherosclerotic and nonatherosclerotic effects may lead to coronary disease. In the present case, coronary computed tomography and IVUS revealed the presence of an atherosclerotic lesion in the anomalous RCA. The IVUS findings also revealed the intramural course of the proximal ectopic artery was elliptical with some lateral compression. The severe stenosis of the intramural course of the proximal ectopic artery was recommended to deploy the stent [9]. However, in the present case, the stenosis of the proximal intramural course was not so severe that we did not deploy the stent in the proximal portion. The patient had no symptoms or signs of ischemia after PCI. The abnormal origin of the RCA may have promoted atherosclerosis development in this patient|.

Coronary revascularization is recommended as class I in patients with RCA originating anomalously and passing between the aorta and pulmonary artery, with evidence of myocardial ischemia [13]. In the present case, the patient needed revascularization because the RCA ran between the aorta and pulmonary artery, with evidence of ischemia on stress radionuclide myocardial perfusion imaging. Coronary bypass surgery is usually selected for such coronary revascularization [8, 13, 14]. However, an increasing number of reports have described the efficacy of PCI in such patients [15]. Because of the unusual anatomical features of anomalous coronary arteries, PCI may be technically challenging. Regarding the selection of the guiding catheter, if the ostium of the anomalous RCA is above the sinotubular junction, an AL-type guiding catheter is likely suitable for PCI. If the ostium of the anomalous RCA is below the sinotubular junction within the left sinus of valsalva, the anomalous RCA will present an acute takeoff angle of the orifice and the vessel course, which was also noted in our patient. Thus, guiding support is poor due to the noncoaxial engagement of most currently available guiding catheters. Successful revascularization in such patients requires adequate techniques such as the deep-seated guiding catheter technique, the buddy wire technique, the anchor balloon techniques, or using a large-caliber guiding catheter, an ST01™ catheter, or a GuideLiner™ catheter with the mother-and-child technique [16–19]. Angelini et al. used dedicated guiding catheters and performed IVUS study successfully [9].

This case illustrates the possibility that the anatomical features of coronary anomalies may promote atherosclerosis and cause angina pectoris, and that PCI may be used for the noninvasive revascularization of anomalous coronary arteries.

## 4. Conclusions

Coronary artery anomalies can cause angina pectoris via both atherosclerotic and nonatherosclerotic effects. Percutaneous coronary angioplasty may be useful for the noninvasive revascularization of atherosclerotic lesions in abnormal coronary arteries.
